# Pharmacognostic and Antioxidant Properties of *Dracaena sanderiana* Leaves

**DOI:** 10.3390/antiox5030028

**Published:** 2016-08-20

**Authors:** Mei Gee Ong, Siti Nur Aishah Mat Yusuf, Vuanghao Lim

**Affiliations:** 1 School of Health Sciences, Health Campus, Universiti Sains Malaysia, Kubang Kerian 16150, Malaysia; meigeeong@gmail.com; 2 Integrative Medicine Cluster, Advanced Medical and Dental Institute, Universiti Sains Malaysia, Kepala Batas 13200, Malaysia; nuraishahyusuf@gmail.com

**Keywords:** *Dracaena sanderiana*, antioxidant, DPPH, Total phenolic content (TPC), Total flavonoid content (TFC)

## Abstract

Endogenous and exogenous antioxidants are used to neutralise free radicals and protect the body from free radicals by maintaining the redox balance. The antioxidant properties of *Dracaena sanderiana* leaves were evaluated using the 2,2-diphenyl-1-picrylhydrazyl (DPPH) assay, and the total phenolic and flavonoid contents were measured. The classes of secondary metabolites were evaluated through pharmacognostic studies, and active compounds were identified by gas chromatography mass-spectrometry (GC-MS). All ethanol-water extracts and *D. sanderiana* leaf powder were positive for tannins, saponins, terpenoids, cardiac glycosides, and quinones. Flavonoids were present in 100%, 80%, 60%, and 40% ethanol extracts (E100, E80, E60, and E40). E100 showed the highest total flavonoid content, whereas E60 extract showed the highest antioxidant activity and total phenolic content. GC-MS revealed the presence of glycerol, 2,3-dihydro-3,5-dihydroxy-6-methyl-(4H)-pyran-4-one, *n*-dodecanoic acid, tetradecanoid acid, (*n*-) hexadecanoid acid, and *n*-octadecanoic acid in the E60 extract.

## 1. Introduction

There has been an expanding interest worldwide on natural product research, especially on medicinal plants which may have restorative properties [1]. Plants are rich with phytochemicals and secondary metabolites that are capable of neutralising free radicals [2,3]. Living organisms produce reactive oxygen species (ROS) as a consequence of normal cellular metabolism. Previous studies showed that ROS cause cell damage which may lead to human diseases [4,5]. Antioxidants are substances that notably inhibit oxidation by neutralising free radicals [6]. Therefore, the development of natural antioxidants has become a focus of study.

Many plants contain compounds that have curative or protective properties against various diseases. Different types of bioactive compounds are responsible for antioxidant activities by neutralising free radicals [7]. *Dracaena sanderiana* (Order Asparagales, Family Asparagaceae), also known as lucky bamboo, is native to tropical West and Central Africa, where it grows up to five feet tall beneath the rainforest canopy. Lucky bamboo is a well-known indoor plant that is considered to be a symbol of good luck [8]. Numerous studies have been conducted on *Dracaena* species, but the antioxidant and pharmacological properties of *D. Sanderiana* have not been reported.

In this study, antioxidant properties, total phenolic contents (TPC), and total flavonoid contents (TFC) of *D. Sanderiana* were evaluated, while the secondary metabolites were analysed through pharmacognostic studies. The active compounds were identified by gas chromatography-mass spectrometry (GC-MS).

## 2. Materials and Methods

### 2.1. Chemicals and Reagents

Methanol (MeOH) and ethanol (EtOH) were supplied by QReC (QRec Asia, Rawang, Malaysia). Folin-Ciocalteu reagent, sodium hydroxide (NaOH), sulphuric acid (H_2_SO_4_), hydrochloric acid (HCl), sodium nitrite (NaNO_2_), glacial acetic acid, chloroform, sodium carbonate (Na_2_CO_3_), aluminium chloride (AlCl_3_), gallic acid, quercetin, butylated hydroxyanisole (BHA), 2,2-diphenyl-1-picrylhydrazyl (DPPH), Dragendorff’s reagent, Mayer’s reagent, and ferric chloride hexahydrate (FeCl_3_·6H_2_O) were supplied by Sigma-Aldrich (Sigma-Aldrich Co, Shanghai, China). All reagents were of analytical grade.

### 2.2. Plant Collection

*D. sanderiana* was collected from Manjung, Perak, Malaysia, from October 2012 until November 2012. The plants were then sent to the Herbarium Unit, School of Biological Sciences, Universiti Sains Malaysia (USM) for botanical identification of the species. The voucher specimen: No. 11432 was deposited in the herbarium.

### 2.3. Extract Preparation

The plants were washed under running tap water and separated into stems and leaves. Freshly collected leaves were chopped into small pieces and oven-dried for one week at 37 °C. The dried plant were homogenised into fine powder using a grinder [9] and subjected to maceration [10] with six different ratios of ethanol-water in a conical flask, 100:0 (E100), 80:20 (E80), 60:40 (E60), 40:60 (E40), 20:80 (E20), and 0:100 (E0) for 24 h in increasing polarity of solvents. Each extract was filtered through a Whatman No. 1 filter paper and concentrated using rotary evaporator (EVELA Rotary Vacuum Evaporator N-1100 Series, Tokyo, Japan) and personal evaporator (GENEVAC EZ-2 Elite Personal Evaporator, Suffolk, UK). The dried extracts were kept at 4 °C in airtight containers until further analysis. The percentage of yield (%) was calculated according to Ayoola et al. [10]. The extract in 70% ethanol was subjected to sonication using an ultrasonicator (MUJIGAE, Seoul, South Korea) for 30 min before testing.

### 2.4. Pharmacognostic Studies

Pharmacognostic screening of *D. sanderiana* extracts for the presence of alkaloids, tannins (FeCl_3_ solution test), phlobatannins, saponins (Foam test), terpenoids (Salkowski test), cardiac glycosides (Keller-Killiani test), and quinine were carried out as described previously [11,12]. The result of each test was qualitatively and phytochemically expressed as negative (−) or positive (+).

### 2.5. Determination of Total Flavonoid Content (TFC)

The TFC in the extracts was measured by the AlCl_3_ method as described by Misra et al. [13] using quercetin as the standard. The flavonoid content was expressed in quercetin equivalents (QE)/g of dry extract (dE). These experiments were run in triplicate.

### 2.6. Determination of Total Phenolic Content (TPC)

TPC in the extracts was measured using the Folin-Ciocalteau method as described by Burgos et al. [14]. The measurement was compared with gallic acid solution as the control. These experiments were run in triplicate. TPC in plant extracts was expressed in gallic acid equivalents (GAE).

### 2.7. DPPH Assay

The free radical scavenging activity of the extract fractions was measured in vitro according to Eniugha et al. [15] using 2,2-diphenyl-1-picryl hydrazyl (DPPH) assay, and the inhibitory concentration (IC_50_) of the extract were estimated and calculated according to the equation:

Radical scavenging activity % = (OD_control_ − OD_sample_)/OD_control_ × 100%

where OD_optical density_ is the absorbance values for control and sample.

### 2.8. Identification of Active Compounds Using GC-MS

The most active fraction, E60, was diluted to 10 mg/mL and analysed using Shimadzu GC-MS QP2010 Ultra system, Kyoto, Japan. The chromatographic separation was performed according to Meena et al. [16]. The data were analysed and compared with the reference of GC-MS library (National Institute of Standard and Technology). Only individual compounds with quality matches >90% based on their percentage of the total area of peaks in the total ion chromatogram were reported.

### 2.9. Statistical Analysis

All experiments were carried out in triplicate. All data were calculated using Excel software (Microsoft Office) and expressed as mean ± standard deviation (SD). The results were analysed by one-way Analysis of Variance (ANOVA) using SPSS software (IBM, Armonk, NY, USA) in order to detect significant difference in six extracts (*p* < 0.05) [9].

## 3. Results and Discussion

### 3.1. Extraction Yield and Pharmacognostic Study

A mixture of water and ethanol was used for extraction, as they offer low toxicity and high extraction yield. Moreover, they can be mixed in different hydroethanolic ratios, which enables the modulation of the polarity of the solvent. Table 1 shows the weight and percentage yield of the E100, E80, E60, E40, E20, and E0 fraction extracts. The E100 had the highest extract yield (4.29 g) and the E20 had the lowest extract yield (0.82 g). Thus, 100% ethanol was the best solvent for extraction.

The pharmacognostic study of *D. sanderiana* leaves showed that both powder and extracts contain a large amount of phytonutrients. The classes of phytochemical groups present are shown in Table 2. All extracts and powder of the plant were positive for tannins, saponins, terpenoids, cardiac glycosides, and quinone. Alkaloids and phlobatannins were absent in the plant.

### 3.2. TFC

Flavonoids have been evaluated in vivo and proven to reduce oxidative stress [17]. The TFC in the plant ethanolic extracts was calculated from the regression equation (*y* = 0.3636*x*, *R^2^* = 0. 9913) of the calibration curve. The TFCs of plant extracts from different solvent ratios ranged from 74.26 to 210.58 mg QE/g dE and differed significantly from each other (*p* < 0.05) (Table 3). E100 was the best solvent for extracting flavonoids from *D. sanderiana* leaves. A high ethanol/water ratio extract does not necessarily contribute to high TFC. This is because flavonoids have different types of structures. Flavonoids with a hydroxyl moiety in the molecule play roles as proton donors and thus exhibit free radical–scavenging activity properties. Moreover, the extracts consist of various bioactive compounds with different specific activities [18].

### 3.3. TPC

Natural phenolics have strong antioxidant properties since these molecules are able to terminate the generation of free radical chain reactions in the presence of hydroxyl groups which act as reducing agents [19]. All phenolic samples, including flavonoids, anthocyanins, and non-flavonoid phenolic compounds, were measured by the Folin-Ciocalteu method [20].

The TPC in the extracts was calculated from the regression equation (*y* = 5.0739 *x*; *R^2^* = 0.9855) of the calibration curve. The TPC of plant extracts ranged from 36.93 to 151.14 mg GAE/g dE (Table 4). E60 had the highest TPC.

A previous study showed that phenolic compounds are directly associated with free radical-scavenging activity [21]. However, the accuracy for the determination of TPC using the Folin-Ciocalteu assay can be affected by various non-phenolic–reducing compounds, for instance organic acid and ascorbic acids, and thus leads to overestimation of the TPC [22]. Moreover, underestimation of the TPC will occur since various types of phenolics react differently with the Folin-Ciocalteu reagent, particularly several phenolic compounds which exhibit low absorption [22].

### 3.4. DPPH

DPPH is a stable nitrogen-centred free radical that acts as an electron acceptor or hydrogen radical. It is reduced to a stable diamagnetic molecule in the presence of an antioxidant molecule and changes colour from purple to yellow. The DPPH assay is a convenient method to evaluate the free radical–scavenging ability of various potent antioxidant sources, especially medicinal plants [23].

In this study the DPPH assay was performed using BHA as the standard. To maintain the stability of free radicals, the DPPH assay was carried out at room temperature [24]. Table 5 shows the percentage inhibition of *D. sanderiana* extracts and BHA. The concentration of various extracts will influence the free radical–scavenging activity [23]. The increasing concentration of the extracts from 0.05 to 1 mg/mL will gradually increase the percentage of inhibition by the DPPH assay as shown in Table 5.

Table 6 shows the IC_50_ values for the extracts. A lower IC_50_ indicates higher antioxidant activities. The E60 extract showed the highest percentage inhibition at 1 mg/mL and the lowest IC_50_ [21].

TFC, TPC, and antioxidant activities of different species of *Dracaena* were evaluated previously. *Dracaena cambodiana* was found to possess a significant amount of TPC and TFC and exhibited antioxidant activity with an IC_50_ of 1.61 mg/mL from ethyl acetate extract [25]. *Dracaena draco* fruit extract showed an IC_50_ of 0.30 mg/mL, lower than the reference used in the study [26]. Shukla et al. reported an IC_50_ value of 0.46 mg/mL for the *Dracaena reflexa* stem dichloromethane extract [27]. *Dracaena umbratica* extracts from leaves, rhizomes, and roots showed antioxidant activities with a low percentage of inhibition [28]. Even though the IC_50_ of E60 of *D. sanderiana* extract was higher compared to *D. draco* fruit extract and *D. reflexa* stem extract, *D. sanderiana* showed a lower IC_50_ value compared to *D. cambodiana*.

Previous studies showed that the presence of a hydroxyl group on the antioxidant molecule will contribute to the reduction mechanism of DPPH and the antioxidant activity of the plant extract [29]. Numerous phytochemicals, such as phenolics, flavonols, carotenoids, and tannins, might contribute to the antioxidant activity of the extracts. In the present study, the extracts from the leaves of *D. Sanderiana* exhibited high antioxidant activity. The E60 extract was the most active fraction, with 91.71% inhibition and an IC_50_ value of 0.5 mg/mL. Moreover, the E60 extract had a comparatively high TPC (151.14 mg GAE/g dE) and a comparatively low TFC (169.48 mg QE/g dE). The results indicated the major antioxidant components might not be flavonoids but might be those other bioactive groups, such as tannins, quinones, cardiac glycosides, saponins, terpenoids, and other phenolic compounds. Furthermore, synergistic effects among the bioactive compounds might affect the high level of antioxidant activity [30]. The presence of glycerol, 2,3-dihydro-3,5-dihydroxy-6-methyl-(4H)-pyran-4-one, *n*-dodecanoic acid, tetradecanoid acid, (*n*-) hexadecanoid acid, *n*-octadecanoic acid, and phenols also might contribute to high antioxidant activity [31,32].

The plant extracts were tested at 1 mg/mL, which is a high concentration for the DPPH assay; however, they did not reach the plateau stage for the percentage inhibition of DPPH. In contrast, the percentage inhibition of DPPH of the BHA standard reached the plateau stage at 0.2 mg/mL, and its IC_50_ was lower than that of the tested extracts. Thus, the results showed that the standard had higher antioxidant activity than the plant extracts. This is because BHA is a pure synthetic antioxidant compound, whereas the plant extract contains mixtures of bioactive compounds. In future studies, the compounds need to be isolated in order to measure the exact antioxidant activity of the plant.

### 3.5. GC-MS Analysis

**Figure 1:**
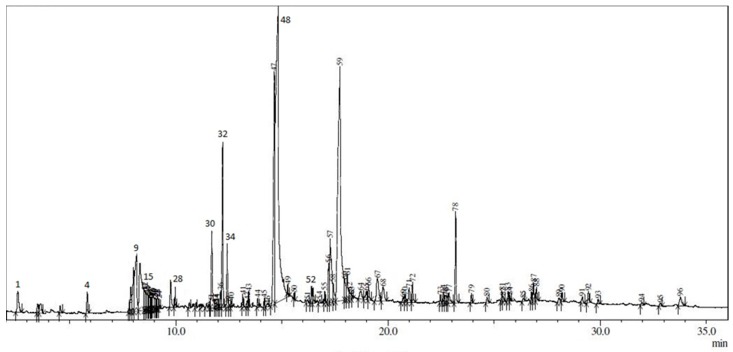
GC chromatogram of E60.

Ninety-six constituents in E60, the most active extract, were identified by GC-MS analysis (Figure 1). The identified compounds with a matching value of 90% and above are reported in Table 7. Several of the identified compounds associated with antioxidant activity have been reported in previous studies. For example, Jerzykiewicz et al. [33] reported that glycerol was found to be a strong antioxidant and Yu et al. [32] showed that 2,3-dihydro-3,5-dihydroxy-6-methyl-(4H)-pyran-4-one has strong antioxidant properties in glucose-histidine Maillard reaction products. Henry et al. [31] reported a few compounds that have high antioxidant activity. For instance, lauric acid (*n*-dodecanoic acid) has 60% antioxidant activity in several food products, such as coconut and soybean products, myristic acid (tetradecanoid acid) has 71% antioxidant activity, and palmitic acid or (*n*-) hexadecanoid acid has 68% antioxidant activity. In contrast, oleic acid has only moderate activity, and stearic acid (*n*-octadecanoic acid) has poor antioxidant activity.

## 4. Conclusions

*D. sanderiana* is a potential source of antioxidants, especially flavonoids and polyphenols. It is important to choose a suitable solvent and process variables to optimise the extraction. The pharmacognostic study of the crude fraction indicated the presence of many phytochemicals, including tannins, flavonoids, saponins, cardiac glycosides, terpenoids, and quinones. TPC, TFC, and DPPH assays were performed to evaluate antioxidant activities. The results showed that *D. sanderiana* leaves have the potential to be used as a natural antioxidant. The E60 fraction had the highest antioxidant activity. GC-MS analysis showed that glycerol, 2,3-dihydro-3,5-dihydroxy-6-methyl-(4H)-pyran-4-one, *n*-dodecanoic acid, tetradecanoid acid, (*n*-) hexadecanoid acid, and *n*-octadecanoic acid were found in the most active fraction, E60. These compounds have been proven to have antioxidant properties in previous studies.

## Figures and Tables

**Table 1:** Weight and percentage yield of crude extract of *D. sanderiana*.

Extract	Weight of Crude Extract (g)	Percentage Yield (%)
E100	6.86	4.29
E80	3.48	2.31
E60	2.76	1.89
E40	3.46	2.45
E20	1.42	0.82
E0	1.69	1.28

**Table 2:** Phytochemical groups of *D. sanderiana* leaves extracts and powder.

Test	Powder	E100	E80	E60	E40	E20	E0
Tannins	+	+	+	+	+	+	+
Phlobatannins	−	−	−	−	−	−	−
Saponins	+	+	+	+	+	+	+
Terpenoids	+	+	+	+	+	+	+
Cardiac glycosides	+	+	+	+	+	+	+
Quinone	+	+	+	+	+	+	+
Alkaloids	−	−	−	−	−	−	−

Key: + Present; − Absent.

**Table 3:** TFC of extracts.

Extracts	TFC (mg QE/g Dry Extract)	*p* < 0.05
E100	210.58 ± 4.28	b, c, d, e, f
E80	177.94 ± 7.36	a, d, e, f
E60	169.48 ± 2.94	a, d, e, f
E40	123.85 ± 7.15	a, b, c, e, f
E20	74.26 ± 1.12	a, b, c, d
E0	76.58 ± 4.60	a, b, c, d

All values are expressed as mean ± SD (*n* = 3). Means with different letters within a row were significantly different (*p* < 0.05). a, E100, b, E80, c, E60, d, E40, e, E20, f, E0.

**Table 4:** TPC of extracts.

Extracts	TPC (mg GAE/g Dry Extract)	*p* < 0.05
E100	138.47 ± 1.95	a, c, d, e, f
E80	148.27 ± 6.32	b, d, e, f
E60	151.14 ± 4.78	a, c, d, e, f
E40	93.47 ± 2.07	a, b, c, d, e, f
E20	58.75 ± 2.00	a, b, c, d, e, f
E0	36.93 ± 0.54	a, b, c, d, e, f

All values are expressed as mean ± SD (*n* = 3). Means with different letters within a row were significantly different (*p* < 0.05). a, E100, b, E80, c, E60, d, E40, e, E20, f, E0.

**Table 5:** Percentage inhibition determined using the DPPH assay.

Concentration (mg/mL)	% Inhibition of Extracts and Standard (BHA)						
	E0	E20	E40	E60	E80	E100	BHA
0.05	0.60 ± 0.18 *	4.18 ± 0.14 *	1.90 ± 0.89 *	3.85 ± 0.80 *	3.54 ± 0.07 *	4.82 ± 0.74 *	3.84 ± 1.49
0.1	0.85 ± 0.06 *	4.16 ± 0.09 *	2.40 ± 0.34 *	4.64 ± 0.11 *	3.75 ± 0.51 *	4.98 ± 0.95 *	27.75 ± 2.53
0.2	0.96 ± 0.10 *	4.17 ± 0.08 *	5.33 ± 0.43 *	22.29 ± 0.51 *	7.86 ± 0.02 *	21.73 ± 0.12 *	91.53 ± 0.18
0.4	3.57 ± 0.41 *	11.51 ± 0.70 *	17.24 ± 0.43 *	34.14 ± 0.19 *	29.21 ± 0.06 *	34.96 ± 0.49 *	93.23 ± 0.62
0.6	5.95 ± 0.53 *	17.68 ± 0.59 *	34.56 ± 0.51 *	74.68 ± 0.07 *	60.36 ± 0.08 *	62.49 ± 0.23 *	93.99 ± 0.62
0.8	8.77 ± 0.53 *	21.43 ± 0.51 *	48.00 ± 0.26 *	84.33 ± 0.30 *	85.35 ± 0.04 *	76.45 ± 0.11 *	94.30 ± 0.57
1.0	11.07 ± 0.80 *	23.50 ± 0.73 *	56.21 ± 0.43 *	91.72 ± 0.42 *	90.21 ± 0.08 *	88.60 ± 0.28 *	94.98 ± 0.07

All values are expressed as mean ± SD (*n* = 3), * *p* < 0.05 indicates a significant difference compared to the standard.

**Table 6:** IC_50_ values of extracts and standard (BHA).

Plant Extracts and Standard	IC_50_ (mg/mL)	*p* < 0.05
E0	4.52 ± 0.38	b, c, d, e, f, g
E20	1.95 ± 0.21	a, d, e, f, g
E40	0.87 ± 0.14	a, d, e, f, g
E60	0.50 ± 0.01	a, b, c, g
E80	0.55 ± 0.01	a, b, c, g
E100	0.54 ± 0.01	a, b, c, g
BHA	0.26 ± 0.01	a, b, c, d, e, f

All values are expressed as mean ± SD (*n* = 3). Means with different letters within a row were significantly different (*p* < 0.05). a, E0, b, E20, c, E40, d, E60, e, E80, f, E100, g, BHA.

**Table 7:** Results of GC-MS analysis conducted to identify compounds from the E60 extract.

Peak	Compound Name	Retention Time (Rt)	Peak Area (%)	Percentage (%)
1	Glycerol	2.55	0.96	92
4	4H-Pyran-4-one, 2,3-dihydro-3,5-dihydroxy-6-methyl	4.55	0.20	92
9	*n*-Dodecanoic acid/Lauric acid	8.15	3.13	96
48	(*n*-) Hexadecanoid acid	14.82	24.48	95
32	Tetradecanoic acid	11.70	2.21	94
52	Nonadecanol	16.39	0.36	95
53	Henicosan-1-ol	16.46	0.47	96
59	Stearic acid	17.74	17.49	95
78	1,2-Benzenedicarboxylic acid, mono(2-ethlhexyl) ester	23.19	2.22	95
34	1-Heptadecene	11.92	0.17	91
61	Amide	18.08	0.91	95
